# Oral Steroid Pulse Therapy for Polymyalgia Rheumatica

**DOI:** 10.7759/cureus.83319

**Published:** 2025-05-01

**Authors:** Shigeko Inokuma, Akio Sasaki, Shun Fukushima, Satoshi Miike, Jun Tamura, Yoshimasa Gotoh, Kazuaki Hara, Takayuki Motoshima

**Affiliations:** 1 Department of Allergy and Rheumatism, Chiba Central Medical Center, Chiba, JPN; 2 Department of Rheumatism and Collagen Diseases, Kohnodai Hospital, National Center for Global Health and Medicine, Chiba, JPN; 3 Department of Internal Medicine, Chiba Central Medical Center, Chiba, JPN; 4 Department of Neurosurgery, Chiba Central Medical Center, Chiba, JPN

**Keywords:** elderly, oral steroid pulse, oral steroid treatment, polymyalgia rheumatica, pulse, pulse steroid therapy, pulse therapy, steroid

## Abstract

Objectives: A new regimen of oral steroid pulse therapy (oral-P), involving intermittent administration of a sufficient oral steroid dose, was adopted for the treatment of polymyalgia rheumatica (PMR). A retrospective evaluation of oral-P in a clinical setting was conducted.

Materials and methods: The medical records of PMR patients, diagnosed according to the American College of Rheumatology/European League Against Rheumatism criteria and treated with oral prednisone (P) between April 2015 and July 2020, were reviewed. One course of oral-P consisted of prednisolone at 0.4 mg/kg/day for three consecutive days, followed by 0.1 mg/kg/day for 11 days (0.4P), or 0.8 mg/kg/day followed by 0.2 mg/kg/day (0.8P). After three or five courses, the dose was tapered. The attending physician selected the treatment regimen. Serum C-reactive protein (CRP) levels and erythrocyte sedimentation rates (ESRs) were monitored. The duration of disease prior to oral-P initiation, follow-up duration after its withdrawal, and any adverse events were assessed.

Results: Thirty-four patients (22 women and 12 men, aged 66-86 years; 15 on 0.4P and 19 on 0.8P; 11/4 on three/five courses with 0.4P, and 13/6 with 0.8P) were included. Prior to oral-P initiation, CRP levels and ESRs were significantly higher in the 0.8P group than in the 0.4P group (CRP: 8.93 (4.83-12.0) vs. 4.96 (4.15-6.31) mg/dL; ESR: 113.5 (92.75-129) vs. 84 (66-95.5) mm/h). Although the disease duration before oral-P initiation was longer in the 0.8P group, the difference was not statistically significant. After the first course, both CRP levels and ESRs decreased significantly in both groups, with no significant differences observed between the groups in subsequent courses. Twenty-one patients were successfully withdrawn from steroid therapy without relapse during a follow-up period of 27 (14-49) months. The remaining 13 patients were still undergoing dose tapering at their last visit, with a median dose of 3 (2-6) mg/day. No severe adverse events were reported.

Conclusion: Oral-P appears to be a promising treatment regimen for PMR, providing rapid symptom relief and enabling eventual steroid withdrawal.

## Introduction

Polymyalgia rheumatica (PMR) is an inflammatory disease that affects the limb girdles, primarily occurring in the elderly [1]. Its prevalence has increased recently due to the aging population [2,3]. Corticosteroids (steroids) have traditionally been the treatment of choice, and long-term use over several years is common [4-6]. Although biologics and JAK inhibitors have recently been introduced, their efficacy and adverse effects still require validation. The American College of Rheumatology (ACR)/European League Against Rheumatism (EULAR) criteria state that PMR typically affects individuals aged 50-60 years or older [7,8]; however, most patients are actually in their 70s or older. Long-term steroid use is undesirable [9]. Nevertheless, achieving sustained remission requires a dose sufficient to suppress PMR pathogenesis. In clinical practice, we adopted novel oral steroid pulse therapy (oral-P) for PMR treatment, which involved a relatively low total cumulative dose. We observed a rapid and favorable response in all patients, with successful treatment withdrawal in most cases.

## Materials and methods

In this study, data obtained in clinical settings were retrospectively examined. The medical records of patients who visited the Chiba Central Medical Center between April 2015 and July 2020, diagnosed with PMR based on the ACR/EULAR criteria [8], and treated with oral-P were retrospectively investigated. These patients were among the first to be evaluated for the disease known as PMR. Records of follow-up examinations up to July 2024 were also reviewed. The Ethical Committee of Chiba Central Medical Center issued approval 2019-R-20 dated December 4, 2019.

One course of oral-P consisted of 0.4 mg/kg/day prednisolone (PSL) for three consecutive days, followed by 0.1 mg/kg/day for 11 days (0.4P), or 0.8 mg/kg/day for three days, followed by 0.2 mg/kg/day for 11 days (0.8P). Three or five courses were administered, after which the dose was tapered by 0.5 mg/day every two weeks for the 0.4P regimen and by 1 mg/day every two weeks for the 0.8P regimen. The attending physician determined the treatment course within the official healthcare system.

Serum C-reactive protein (CRP) levels and erythrocyte sedimentation rates (ESRs) were collected at the first visit, at the initiation of oral-P treatment, after each course, and upon completion of all courses. The Mann-Whitney U test was used to compare the 0.4P and 0.8P groups, while the paired t-test was used for within-group comparisons across time points. A p-value of less than 0.05 was considered statistically significant. As the distributions of CRP levels and ESRs at each time point were right-skewed, logarithmic transformation was performed prior to the paired t-test.

MRI and/or ultrasonographic findings of the shoulder girdles and ophthalmological findings were evaluated. The presence of autoantibodies was also examined. Previous medications and drugs prescribed concurrently with oral-P were documented. Medical records were reviewed to assess rebound and significant adverse events.

As the treatment was implemented in routine clinical practice under the healthcare system, informed consent was not obtained. Data cannot be shared openly to protect patient privacy.

The cumulative PSL dose and dosing period for each standard oral-P course were calculated. For comparison with the conventional treatment protocol, the estimated cumulative PSL doses described in one report and two recommendations [4,7,8] were also calculated.

## Results

Thirty-four patients (22 women and 12 men; age range: 66-86 years) were included in this study (Table 1). They were selected from among 59 patients diagnosed with PMR. The remaining 25 patients were treated with one, two, or all of the following: ibuprofen, colchicine, and methotrexate (MTX). The treatment choice was made by the attending physician in the clinical setting. Fifteen patients (eight women and seven men; age range: 67-86 years) received 0.4P, and 19 patients (14 women and five men; age range: 66-85 years) received 0.8P. The body weights were 58.0 kg (interquartile range: 47.7-63.7 kg) in the 0.4P group and 54.0 kg (interquartile range: 51.5-56.6 kg) in the 0.8P group. In the 0.4P group, three courses were administered to 11 patients, and five courses were administered to four patients. In the 0.8P group, three and five courses were administered to 13 and six patients, respectively.

**Table 1:** PMR patients treated with oral-P 0.4P, 0.4 mg/kg oral-P; 0.8P, 0.8 mg/kg oral-P p-value, by Mann-Whitney U test comparing values of 0.4 vs. 0.8 groups p-value, by paired t-test comparing values at introduction and after each course, using log-transformed CRP and ESR CRP: C-reactive protein, ESR: erythrocyte sedimentation rate, oral-P: oral steroid pulse therapy, IQR: interquartile range

	0.4P				0.8P				p-value 0.4P vs. 0.8P
n (female/male)	15 (8/7)				19 (14/5)				0.232
	Median	IQR	n	p-value vs. introduction	Median	IQR	n	p-value vs. introduction	p-value 0.4P vs. 0.8P
Age	80	76–81.5	15	-	77	75.5–78.5	19	-	0.045
Since disease onset (days)	30	19–41.5	15	-	40	30–62	19	-	0.063
CRP (mg/dL)									
At first visit	6.26	3.32–7.16	15	-	7.99	4.58–12.6	19	-	0.061
At oral-P introduction	4.96	4.15–6.31	15	-	8.93	4.83–12.0	19	-	0.020
After 1st course	1.8	0.8–2.40	15	0.001>	1.25	0.41–3.35	19	0.001>	0.755
After 2nd course	0.9	0.66–1.44	15	0.001>	1.03	0.24–2.73	19	0.001>	0.768
After 3rd course	0.75	0.49–1.22	15	0.001>	0.49	0.28–2.59	19	0.001>	0.876
After 4th course	0.34	0.17–0.54	4	0.004	0.955	0.15–2.18	6	0.024	0.205
After 5th course	0.575	0.43–0.67	4	0.013	0.89	0.32–1.59	6	0.018	0.176
At last visit	0.23	0.10–0.62	15	0.001>	0.25	0.14–0.47	19	0.001>	0.903
ESR (mm/h)									
At first visit	84	66–95.5	15	-	97	89–130	19	-	0.011
At oral-P introduction	79	65–102.5	15	-	113.5	92.75–129	19	-	0.007
After 1st course	37	28–61.5	15	0.001>	51	27–88	19	0.001>	0.107
After 2nd course	36	17–40	15	0.001>	27	12–48	19	0.001>	0.945
After 3rd course	21	18–34.5	14	0.001>	26	12.75–42.2	18	0.001>	0.822
After 4th course	9.5	8.25–11.5	4	0.001	21.5	19.25–23.7	6	0.002	0.145
After 5th course	10.5	9–12	4	0.001>	18.5	15–24.2	6	0.015	0.145
At last visit	12	10–31	13	0.001>	26	19-35	17	0.001>	0.290

MRI and echography of the shoulder girdles were performed on 26 and 14 patients, respectively. In all cases, the findings were consistent with bursitis. Five patients did not undergo either of the imaging modalities. None of the patients had crystal arthritis. One male patient in the 0.8P group presented with jaw claudication, and echography revealed thickening of the entire circumference of the bilateral temporal arteries. He was subsequently diagnosed with temporal arteritis. All patients underwent ophthalmological examinations, which showed no ocular involvement. No autoantibodies (RF, anti-CCP antibody, ANA, anti-SS-A/Ro antibody, anti-SS-B/La antibody, PR3-ANCA, or MPO-ANCA) were detected in any of the patients.

As prescribed by their previous physicians, patients had taken various nonsteroidal anti-inflammatory drugs, including celecoxib (20 patients), loxoprofen (five patients), diclofenac (two patients), acetaminophen and tramadol (two patients), sulindac (one patient), and lornoxicam (one patient). These medications had proven ineffective. After visiting our hospital, ibuprofen (600 mg/day) was administered to 21 patients, and 1 mg of colchicine was added to the ibuprofen regimen in 10 patients prior to oral presentation. MTX was subsequently added to oral-P in 15 patients (two in the 0.4P group and 13 in the 0.8P group), of whom eight received it after oral-P had already taken effect. The weekly MTX dose was 8 mg (range: 5.5-9.5 mg), and it was later withdrawn in three patients.

**Figure 1:**
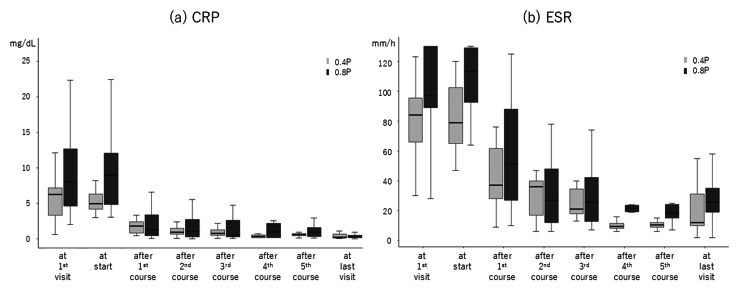
Serum CRP level (a) and ESR (b) during the courses of oral-P Serum CRP level (a) and ESR (b) during the courses of oral-P with 0.4 mg/kg/day PSL (0.4P, light) and 0.8 mg/kg/day PSL (0.8P, dark). Either from the first visit or the start of oral-P to the end of the first course and thereafter, the CRP level and ESR significantly decreased in both groups (p<0.05). CRP: C-reactive protein (mg/dL), ESR: erythrocyte sedimentation rate (mm/h), oral-P: oral steroid pulse therapy, PSL: prednisolone

The durations from clear disease onset to oral-P initiation were 30.0 days (19.0-41.5) in the 0.4P group and 40.0 days (30.0-62.0) in the 0.8P group (p=0.063) (Table 1). Serum CRP levels and ESRs are shown in Table 1 and Figure 1a-1b. At their first visit, the CRP level (normal range: <0.3 mg/dL) was higher, though not significantly so, and the ESR (normal range: <20 mm/h) was significantly higher in the 0.8P group compared to the 0.4P group. At the time of oral-P initiation, both the CRP level and ESR were significantly higher in the 0.8P group (8.93 (4.83-12.0) vs. 4.96 (4.15-6.31) mg/dL; 113.5 (92.75-129) vs. 84 (66-95.5) mm/h). After the first oral-P course, the CRP level and ESR no longer differed significantly between the two groups (Table 1). In both groups, the CRP level and ESR decreased significantly from their initial visit values or the values at oral-P initiation (Table 1, Figure 1a-1b).

Twelve patients from the 0.4P group and nine patients from the 0.8P group were successfully withdrawn from the oral-P course. No relapses occurred in these 21 patients. At a median follow-up of 27 months (range: 14-49 months) after withdrawal, they showed CRP levels below the upper limit of normal and ESRs ranging from 2 to 46 mm/h. The remaining 19 patients, who were still undergoing PSL dose tapering, showed no relapse at their last follow-up, with a PSL dose of 3 mg/day (range: 2-6 mg/day).

The observed adverse events included elevated blood sugar levels in three patients, urinary tract infections in two patients, gastric ulcers in one patient, elevated intraocular pressure in another patient, osteoporosis in a third patient, and suspected enteritis in a fourth patient. These adverse events were not severe, and acute events were appropriately managed. None of the patients discontinued treatment before completing their course, and no changes were made to their dosing schedule.

Under the oral-P protocol used in this study, the estimated total cumulative PSL doses until withdrawal completion were 660 mg and 890 mg for three and five courses of 0.4P, and 1320 mg and 1780 mg for three and five courses of 0.8P, respectively, assuming a body weight of 50 kg. When considering a weight of 60 kg, the cumulative doses were 960 mg and 1152 mg (0.4P, three and five courses), and 1320 mg and 2304 mg (0.8P, three and five courses), respectively. In the 0.4P and 0.8P protocols, the dosing periods were estimated to be 24 and 28 weeks (for a 50 kg patient, with three and five courses, respectively) and 28 and 32 weeks (for a 60 kg patient, with three and five courses, respectively).

In contrast, a conventional treatment protocol starts with a PSL dose of 15 mg, regardless of body weight, with dose tapering initiated at three weeks [4]. The cumulative dose over 80 weeks under this protocol is estimated to be 3265.5 mg. According to the ACR/EULAR-recommended protocol [7], the initial PSL dose is 15 mg, with an estimated cumulative dose of 1662.5 mg over 25 weeks prior to tapering. In the revised ACR/EULAR recommendation [8], the initial PSL dose ranges from 12.5 to 25 mg, and the estimated cumulative doses until withdrawal completion range from 1890 to 2695 mg over a minimum treatment period of 12 months.

## Discussion

The oral-P therapy is a novel approach that uses intermittent dosing. This study presents a substantial number of cases treated with oral-P. Regarding the background of introducing oral-P for PMR, we have long used oral-P for other inflammatory diseases, not just PMR. We observed that during standard intravenous methylprednisolone pulse therapy, a low oral dose administered after three days of high-dose therapy did not cause any issues related to dose reduction. On the other hand, a sufficient dose suppresses disease pathogenesis within three days, even when administered intermittently. We considered that in cases where a nongenomic effect, achievable only at extraordinarily high doses, is not required, but only a sufficient genomic effect is needed, the genomic dose could also be given intermittently. Based on this reasoning, we introduced an oral steroid pulse regimen for PMR treatment. The aim is to satisfactorily suppress PMR pathogenesis with a sufficient genomic steroid dose while avoiding adverse events associated with continuous administration.

Steroids are the principal treatment for inflammatory diseases, with doses ranging from low oral doses to high-dose intravenous pulse therapy. Oral-P begins with a conventional medium dose (0.4 or 0.8 mg/kg) of PSL for three consecutive days. Currently, the recommended daily PSL dose for PMR is up to 25 mg, and initial doses exceeding 30 mg/day are strongly discouraged. Considering the smaller body size of Japanese patients, either oral-P dose would be high enough to satisfactorily induce a disease-modifying effect.

In this study, even though a low dose was used for three days following a medium dose, the first course elicited a prompt effect, demonstrated by a substantial decrease in CRP levels and ESR (CRP/ESR). The paired t-test in this analysis simply compared pre- and post-treatment values and does not distinguish between regression to the mean and true treatment effects. Thus, a strict causal relationship between the treatment and reductions in CRP/ESR cannot be claimed. However, given the exploratory nature of the study and the clinical understanding that PMR rarely undergoes spontaneous remission in a short period, the observed rapid decrease in CRP/ESR is considered suggestive evidence of treatment efficacy and provides clinically valuable insight.

Twenty-one patients were completely withdrawn from oral-P, including those with follow-up of over a year. The remaining 13 patients were undergoing further dose tapering, already reduced to as low as 2-6 mg/day of PSL. The adverse events observed were typical of steroid therapy and were not severe. Although the patients ranged in age from 60 to 80 years, none required changes to their dosing schedule.

This was a retrospective study, not a prospective one. The choice of the oral-P course was made by the attending physician in a clinical setting and was not randomized; thus, exchangeability is not guaranteed. Subsequent analysis revealed that the 0.8P group had significantly higher baseline CRP/ESR levels and longer disease durations than the 0.4P group prior to the introduction of oral-P. However, these differences did not reach statistical significance. An earlier introduction of therapy for less severe cases may have led to the choice of 0.4P. The lack of a significant difference in CRP/ESR after oral-P suggests that an appropriate selection based on disease severity can be effective, regardless of initial disease activity. With proper case selection, complete withdrawal could be expected with either the 0.4P or 0.8P regimen.

The ibuprofen dose prescribed before oral-P introduction was 600 mg/day, which is the typical dose in Japan and lower than standard doses in Western countries [10]. In some cases, despite administering ibuprofen and colchicine, CRP levels and ESR remained high, prompting the introduction of oral-P (Table 1). MTX was administered to fewer than half of the patients after oral-P was started. Concerns about prolonged steroid use likely led to the addition of MTX. Although some patients received these medications, only oral-P was fully effective, with recovery observed immediately after it was initiated.

Regarding the estimated cumulative steroid dose, it might be 3265.5 mg at 80 weeks [4], 1662.5 mg before tapering [7], and 1890-2695 mg over a minimum of 12 months [9]. In contrast, under the oral-P protocol used in this study, even assuming five courses of 0.8P for a 60-kg patient, the total cumulative dose would be 2304 mg over 32 weeks. Although these are estimates, the oral-P protocol could achieve a lower cumulative dose, shorter treatment duration, and successful complete withdrawal.

The mechanism of action of intravenous methylprednisolone pulse therapy is considered different from conventional therapy. The former activates a nongenomic pathway, which is only achievable at high doses and may be achieved via intravenous administration [11]. The oral-P doses used in this study, administered over three consecutive days, were much lower than those used in intravenous pulse therapy. However, to improve PMR with orally administered steroids, our dose was sufficient to produce noticeable effects after the first pulse. The prompt relief and successful withdrawal seen with oral therapy were likely achieved via the genomic pathway. Although the 0.4P dose might not be a full oral dose, it was considered adequate by the attending physicians for patients with lower CRP levels and shorter disease durations. Traditionally, clinicians may have avoided full-dose oral therapy for PMR due to concerns about glucocorticoid-related adverse events in the elderly. This hesitation could lead to prolonged treatment at insufficient doses, increasing the risk of relapse [12]. In our study, a sufficient effect was achieved with an adequate oral dose, without serious adverse events, and complete withdrawal was successful.

This study only reviewed the medical records of patients treated with oral-P. A clinical trial with a control group is needed to demonstrate the benefits of oral-P. If a cut-off CRP level, ESR, or duration from disease onset to oral-P initiation can be identified through a comparative study of 0.4P and 0.8P in a larger population, an optimal regimen could be more precisely determined.

Oral-P may have broader indications for other steroid-responsive diseases. When a sufficient disease-modifying steroid dose can be prescribed, long-term maintenance doses may no longer be necessary. Even in cases where oral-P is ineffective, it can be promptly withdrawn without the complications associated with long-term use.

The most important contribution of this study is the introduction of the oral-P regimen. However, a key limitation is that this was a retrospective observational study.

## Conclusions

A novel oral-P therapy was introduced for the treatment of PMR. All 34 patients experienced prompt relief. Twenty-one patients were completely withdrawn from the therapy, while the others were undergoing dose tapering. No relapses or severe adverse events were observed. The estimated cumulative dose and dosing period were both lower for oral-P compared to conventional steroid therapy. Oral-P appears to be a promising treatment regimen for PMR, a condition to which the elderly are particularly susceptible.
